# The hunt for hidden hearing loss in humans: From preclinical studies to effective interventions

**DOI:** 10.3389/fnins.2022.1000304

**Published:** 2022-09-15

**Authors:** Joaquin T. Valderrama, Angel de la Torre, David McAlpine

**Affiliations:** ^1^National Acoustic Laboratories, Sydney, NSW, Australia; ^2^Department of Linguistics, Macquarie University Hearing, Macquarie University, Sydney, NSW, Australia; ^3^Department of Signal Theory, Telematics and Communications, University of Granada, Granada, Spain; ^4^Research Centre for Information and Communications Technologies (CITIC-UGR), University of Granada, Granada, Spain

**Keywords:** speech-in-noise hearing difficulties, cochlear synaptopathy, central gain, demyelination, noise-induced hearing loss, noise exposure, hearing aids, hearables

## Abstract

Many individuals experience hearing problems that are *hidden* under a normal audiogram. This not only impacts on individual sufferers, but also on clinicians who can offer little in the way of support. Animal studies using invasive methodologies have developed solid evidence for a range of pathologies underlying this *hidden* hearing loss (HHL), including cochlear synaptopathy, auditory nerve demyelination, elevated central gain, and neural mal-adaptation. Despite progress in pre-clinical models, evidence supporting the existence of HHL in humans remains inconclusive, and clinicians lack any non-invasive biomarkers sensitive to HHL, as well as a standardized protocol to manage hearing problems in the absence of elevated hearing thresholds. Here, we review animal models of HHL as well as the ongoing research for tools with which to diagnose and manage hearing difficulties associated with HHL. We also discuss new research opportunities facilitated by recent methodological tools that may overcome a series of barriers that have hampered meaningful progress in diagnosing and treating of HHL.

## Introduction

The World Health Organization (WHO), in its “2021 World Report on Hearing,” estimates that half the global population is at risk of developing hearing loss due to unsafe listening practices (World Health Organization, 2021), including exposure to loud sounds at work and during social activities. Up to 1/3 of the workforce is regularly exposed to damaging levels of loud sounds (Schneider, 2005), and more than half of people aged 12–35 regularly expose themselves to sound levels that pose a risk to hearing either from personal listening devices or by attending loud venues such as nightclubs (Sliwinska-Kowalska and Zaborowski, 2017).

Early signs of hearing loss usually involve difficulties understanding speech in noisy environments, often with no discernible change in hearing thresholds (Lopez-Poveda, 2014; Bramhall et al., 2019). This form of hearing problem is widely referred to as hidden hearing loss (HHL, Schaette and McAlpine, 2011)—*hidden* because it is not possible to diagnose using best-practice clinical tools, such as the audiogram (Bramhall et al., 2019). In fact, one in ten patients who visit a hearing clinic reporting speech-in-noise difficulties remain untreated because the nature of their hearing difficulties cannot be determined (Pryce and Wainwright, 2008; Tremblay et al., 2015; Parthasarathy et al., 2020).

It is now well accepted that hearing loss negatively impacts mental health, behavior, and quality of life, and increases the risk of social isolation, anxiety and depression (Pryce and Wainwright, 2008; Tremblay et al., 2015). Alarmingly, hearing loss in midlife represents the single largest modifiable risk factor for a later dementia diagnosis (Ford et al., 2018; Livingston et al., 2020). Similar assessments of the impacts of HHL on broad health outcomes are now underway. Using *design thinking* methodologies based on online surveys and semi-structured interviews Mealings et al. (2020) reported unmet needs from individuals experiencing HHL and from the clinicians who treat them. They showed that individuals with HHL report that hearing difficulties severely impacted their quality of life, leading them to expend more effort in, and receive less enjoyment from everyday conversations. The same people also reported that missing information in conversations provoked frustration and anxiety associated with potentially misinterpreting what was said. These hearing problems led them to significantly curtail their social encounters. Clinicians reported that they had insufficient training or resources to support such individuals, and that they lacked confidence when recommending treatment options. The main reason for this lack of confidence was the absence of any sensitive measure with which to diagnose HHL; and no uniform or standardized, evidence-based protocol to diagnose and treat their patients.

Considering the potentially high incidence of HHL (Pryce and Wainwright, 2008; Tremblay et al., 2015), its impacts on every-day communication (Ford et al., 2018; Livingston et al., 2020; Mealings et al., 2020), the absence of standardized clinical protocols (Bramhall et al., 2019; Mealings et al., 2020), and the high risk for progression to more severe hearing difficulties (Schneider, 2005; World Health Organization, 2021), there is an urgent need to improve the diagnosis of HHL and to offer solutions to clinicians and their patients.

Here, we review specific highlights of the state-of-the-art relative to the diagnosis and management of HHL (sections “Diagnosing *hidden* hearing loss” and “Intervention strategies for *hidden* hearing loss,” respectively), and discuss perspectives and forthcoming trends enabled by emerging methodological tools and outcomes—some of them developed by our own laboratories (section “Discussion”). For clarity, this paper uses the term “HHL” according to the definition provided by the WHO—<<*the condition where an individual experiences common symptoms associated with noise-related auditory damage, such as difficulty in hearing in noise, and that is undetectable on pure-tone audiometry*>> (World Health Organization, 2021).

## Diagnosing hidden hearing loss

### Neurophysiological pathologies in animal models

Neurodegeneration induced by aging and over-exposure to loud sounds is considered a contributing factor in those who struggle to understand speech, particularly in environments with high levels of background noise. Evidence suggests that at least four neurophysiological pathologies impair the encoding of sounds without elevating hearing thresholds. These, likely related, and potentially interactive, pathologies are cochlear synaptopathy, auditory nerve demyelination, elevated neural gain in the central nervous system, and impaired neural adaptation.

The concept of *cochlear synaptopathy* was first posited by Kujawa and Liberman (2009), who reported that mice experiencing a single exposure to octave band noise (8—16 kHz) at 100 dB sound pressure level (SPL) for 2 h showed an acute and irreversible loss of synaptic ribbons (specialized structures in cochlear sensory hair cells responsible for the release of neurotransmitter required to generate action potentials in afferent auditory nerve fibers) and a subsequent degeneration of these fibers in the absence of any obvious damage to the sensory hair cells. The general tenet of these findings—since replicated in a range of mammalian species, including guinea pigs (Lin et al., 2011; Furman et al., 2013), rats (Bing et al., 2015; Niwa et al., 2016), mice (Chambers et al., 2016; Maison et al., 2016), gerbils (Bourien et al., 2014; Gleich et al., 2016), and rhesus monkeys (Valero et al., 2017)—is that high-threshold auditory nerve fibers (i.e., those with high threshold for sound-evoked activity) are more vulnerable to the damaging effects of loud sounds (noise exposure) than are low-threshold fibers (Furman et al., 2013; Liberman et al., 2015). In addition to over-stimulation, cochlear synaptopathy is also thought to be the primary neural degeneration in age-related hearing loss (Sergeyenko et al., 2013; Kujawa and Liberman, 2015). These findings are consistent with the notion that both aging and noise exposure impact directly the neural encoding of sounds at suprathreshold levels, and suggest that cochlear synaptopathy underlies difficulties understanding speech in noise in individuals with otherwise normal audiograms.

**FIGURE 1:**
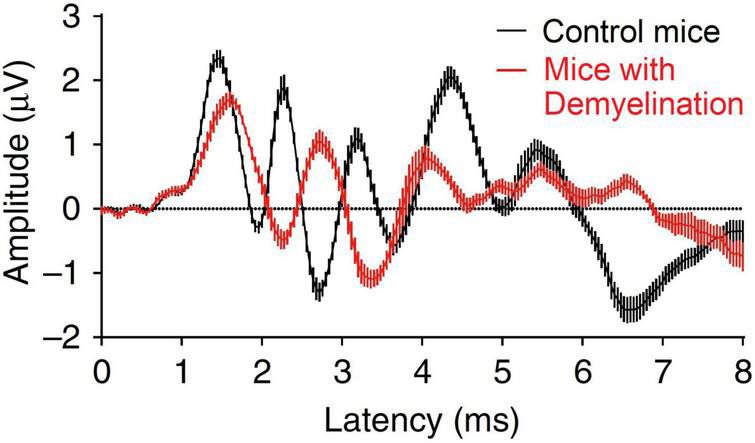
Effect of demyelination on the mice auditory brainstem response. Figure adapted from Wan and Corfas (2017).

A second potential contributing factor to HHL within the inner ear is *auditory nerve demyelination*, a pathology that results from an inefficient repair that follows a loss of cochlear Schwann cells in peripheral terminals of Type I spiral ganglion neurons (Wan and Corfas, 2017). Auditory nerve demyelination occurs independent of noise exposure and is therefore potentially additive to the effects of cochlear synaptopathy. The morphology of sound-evoked auditory brainstem responses (ABRs, Figure 1) suggests that demyelination reduces the neural synchrony of the auditory nerve, evident as a reduction in the amplitude and an increase in latency of ABR wave I, and an increase in neural transmission time from cochlea to the cochlear nucleus, assessed in terms of the difference in latency between the first two peaks of the ABR (Wan and Corfas, 2017). Impaired processing following demyelination might also be expected to impact the precise timing required for successful spatial hearing (Stange-Marten et al., 2017), leading to impaired speech-in-noise performance (Swaminathan et al., 2016). Interestingly, the apparently permanent nature of this pathology might also explain hearing problems that arise in those who suffer acute demyelinating diseases such as Guillain-Barré syndrome (Nelson et al., 1988; Takazawa et al., 2012).

Beyond direct effects on auditory nerve fibers, *in vivo* studies have shown that reduced cochlear output arising from cochlear synaptopathy triggers a series of changes in neural processing in later stages of the auditory system that may explain some of the reported manifestations of HHL in humans (Schaette and McAlpine, 2011; Bakay et al., 2018; Resnik and Polley, 2021); specifically, elevated central-gain and mal-adaptation to unfolding sound environments.

**FIGURE 2:**
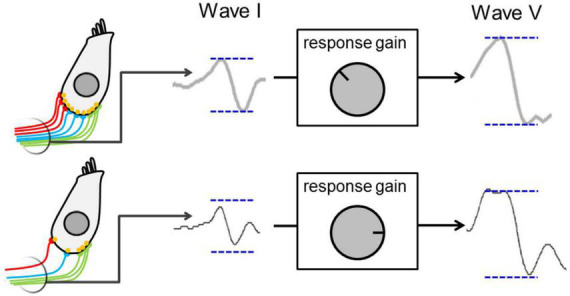
Schematic presenting a model for elevated central gain—decreased auditory nerve activity resulting from cochlear synaptopathy leads to a lower wave I amplitude and to activation of central-gain mechanism that increase neural sensitivity and restore wave V amplitude at the level of the midbrain. Figure adapted from Schaette and McAlpine (2011).

*Elevated central gain* refers to a (potentially homeostatic) increase in neural sensitivity (or activity) in the central auditory system, arising as early as the cochlear nucleus in the brainstem (Schaette and Kempter, 2006), and evident in the midbrain nucleus of the inferior colliculus (Schaette and McAlpine, 2011; Auerbach et al., 2014; Hesse et al., 2016; Monaghan et al., 2020) and auditory cortex (Resnik and Polley, 2021). Figure 2 presents a schematic model of the central-gain hypothesis at the level of the midbrain (Schaette and McAlpine, 2011), in which elevated central gain arises from reduction in excitatory that generates a, potentially compensatory, change in the balance of excitatory and inhibitory neural activity in an attempt to restore the neural representation of sound following some form of cochlear insult, e.g., denervation through cochlear synaptopathy (Schaette and McAlpine, 2011; Auerbach et al., 2014). Whilst this compensatory mechanism helps restore sounds detection in quiet, it impairs the neural representation of speech and impacts temporal processing of sounds in background noise (Chambers et al., 2016; Monaghan et al., 2020; Resnik and Polley, 2021). Interestingly, Hesse et al. (2016) showed that elevated central gain was more pronounced in animals with synaptopathy (exposed to 100 dB SPL noise) than in animals with a permanent increase in hearing threshold (exposed to 105 dB SPL noise), thus suggesting a non-monotonic relationship between subtle cochlear damage and elevated central gain. In addition, elevated central gain may contribute to pathologies such as tinnitus or hyperacusis (Schaette and McAlpine, 2011; Auerbach et al., 2014; Hesse et al., 2016). It is worth noting that the minimal requirement for elevated neural gain may simply be a reduction in sensory input brought about by reduced sound levels or conductive forms of hearing loss (Maslin et al., 2013; Munro and Turtle, 2013; Parry et al., 2019).

*Neural mal-adaptation* refers to the inability of neurons along the auditory pathway to adapt their response to loud acoustic environments to optimize the neural encoding of information in those environments (Dean et al., 2005)—potentially critical for understanding speech in noise. Dean et al. (2008) demonstrated that neurons in the auditory midbrain of guinea pigs adapt their firing pattern to the mean sound level of the background with the consequence that sensitivity to those sound levels improves over time. This form of neural adaptation, evident in the responses of auditory nerve fibers (Wen et al., 2009), is expanded by the level of auditory cortex (Watkins and Barbour, 2008), and is altered in HHL. Specifically, Bakay et al. (2018) found that the ability of midbrain neurons to adapt to loud sound environments was impaired in mice with noise-induced synaptopathy, relative to control mice with no prior noise exposure. This supports the view that hearing-in-noise difficulties in humans might arise from suboptimal neural adaptation to loud sound environments.

### Candidate measures of hidden hearing loss

An important methodological challenge to diagnosing the pathologies that underlie HHL in living humans is the lack of potential biomarkers [i.e., biological marker—an externally measurable representation of a specific condition or pathology (Strimbu and Travel, 2010)] of inner ear physiology and anatomy that mirror invasive methodologies in *in-vivo* animal preparations such as immunostaining or serial-section electron microscopy (Viana et al., 2015). To this end, current diagnostic tools for assessing HHL continue to rely on non-invasive methodologies commonly employed in assessing hearing function.

The most widely reported measure is the amplitude of the click-evoked wave I of the ABR, measured at suprathreshold sound levels. Kujawa and Liberman (2009) reported that the suprathreshold increase in magnitude of ABR wave I with increasing sound intensity was correlated with the number of intact synapses in the auditory nerve following noise injury in rodents, with a lower rate of increase associated with evidence of cochlear synaptopathy. In human listeners, the ratio of the amplitude of waves I and V of the click-evoked ABR has been proposed as an indicator of elevated central gain—a relative measure within the individual that is intended to reduce inter-subject variability and is based on the hypothesis that cochlear synaptopathy generates a reduced amplitude wave I and a compensatory increase in wave V amplitude in audiometrically normal individuals with tinnitus (for whom the term HHL was originally coined; Schaette and McAlpine, 2011). Further, Mehraei et al. (2016) reported that, relative to control animals, noise-exposed mice showed a shorter shift in latency of ABR wave IV (equivalent to wave V in humans) with increasing levels of masking noise—a result consistent with the selective loss of high-threshold auditory nerve fibers expected in individuals with HHL (Bourien et al., 2014). Together, the data are consistent with the relative magnitude of ABR waves being a potential biomarker of HHL.

Liberman et al. (2016) hypothesized that the ratio of amplitude of the summating potential and the compound action potential (SP/AP) amplitude ratio might also represent a biomarker sensitive to HHL. Since cochlear synaptopathy affects the auditory nerve synapses but leaves the cochlear sensory hair cells intact (Kujawa and Liberman, 2009), higher scores of this indicator are expected to be associated with cochlear synaptopathy. Consistent with their hypothesis, Liberman et al. (2016) found that the amplitude ratio of the SP/AP was higher individuals at high risk for ear damage, characterized by normal hearing thresholds up to 8 kHz but elevated thresholds over the extended, high-frequency range (up to 16 kHz). However, counter to their hypothesis, the greater amplitude ratio of the SP/AP in the high-risk group was associated with a higher magnitude SP, rather than a reduction in the magnitude of the AP, making it difficult to interpret in terms of potential synaptopathy. Wan and Corfas (2017) showed that, relative to controls, animals with confirmed auditory nerve demyelination showed similar SP amplitudes but reduced AP amplitudes, with a concomitant increase in the SP/AP ratio. While these results potentially support this measure acting as biomarker for HHL, the fact that the SP is generated by multiple sources, not only inner-hair cells, but also outer-hair cells and even the auditory nerve (Durrant et al., 1998; Pappa et al., 2019; Lutz et al., 2022), renders its use as a biomarker for HHL unlikely.

The envelope following response (EFR) is an auditory steady-state response (i.e., a periodic neurophysiological response resulting from the sum of several overlapping auditory evoked potentials—usually analyzed in the frequency domain; Valderrama, 2022) evoked by an amplitude-modulated tone, and has been used as a physiological measure of the temporal representation of suprathreshold sounds in the auditory brain (Bharadwaj et al., 2014). EFR amplitudes appear smaller in mice with noise-induced synaptopathy, relative to unexposed control mice (Shaheen et al., 2015). Consistent with this, Bharadwaj et al. (2015) reported a significant correlation between the slope of the EFR magnitude as a function of modulation depth and amplitude-modulation detection threshold—a behavioral measure of temporal coding—in normal-hearing young adults. Further, Parthasarathy et al. (2020) found that the combination of ASSR to frequency modulation, pupillometry measures, and a behavioral measure based on a frequency-modulation (FM) detection task accounted for 78% of the speech-perception variability in adults with hearing thresholds in the normal range. However, the relationship of this measure of FM detection to HHL remains complex since, over the near-normal hearing range, sensitivity to slow-FM (a proposed metric for HHL) is correlated with place-coding fidelity (i.e., variations in the cochlear place of stimulation), a likely consequence of “standard” hearing loss arising from damage to cochlear hair cells, rather than retro-cochlear damage (Whiteford et al., 2020).

Vander Ghinst et al. (2021) used magnetoencephalography to record cortical responses to the envelope of running speech in multi-talker background noise, and found that individuals with normal audiograms but difficulties understanding speech-in-noise showed reduced cortical tracking of speech, relative to control individuals who did not have hearing difficulties. This is consistent with the degraded neural representation of speech in background noise observed in noise-exposed animals (Monaghan et al., 2020). Vander Ghinst et al. (2021) also found that human listeners with hearing difficulties showed an increased functional connectivity between auditory cortices and brain areas involved in semantic and attention processes, consistent with Yeend et al. (2017) who reported that selective attention was a significant predictor of speech-in-noise problems in many individuals with presumed HHL.

Finally, the middle-ear muscle reflex (MEMR) has been proposed as a potential biomarker of cochlear synaptopathy due to its strong dependence on the integrity of high-threshold auditory nerve afferent fibers (Liberman, 1988; Kobler et al., 1992). Loud sounds contract the stapedius muscle, stiffening the ossicular chain and tilting the stapes away from the cochlea. This elicits a bilateral increase in middle-ear impedance that can be assessed by measuring otoacoustic emissions (Boothalingam and Goodman, 2021). Valero et al. (2016, 2018) found that MEMR thresholds were elevated, and suprathreshold amplitudes attenuated in noise-exposed mice, relative to unexposed animals. Consistent with these data, Wojtczak et al. (2017) showed that normal or near-normal hearing individuals with tinnitus presented a significantly weaker MEMR strength, compared to individuals without tinnitus. However, these results were not replicated by Guest et al. (2019), who found no association between the MEMR threshold and tinnitus, speech-in-noise hearing performance or noise exposure history in individuals with normal audiograms.

### Sensitivity of candidate measures

Despite the large number of non-invasive candidate measures potentially sensitive to HHL in humans, there is no consensus view that the neurophysiological pathologies evident in animal models of HHL are evident in humans or that these represent the underlying cause of speech-in-noise hearing difficulties reported by individuals with normal audiograms (Kobel et al., 2017; Barbee et al., 2018; Bramhall et al., 2019; Kohrman et al., 2020; Bramhall, 2021).

A possible argument explaining the differences in outcomes across studies and null results across the literature is that the human auditory structures are less susceptible to the adverse effects of noise exposure than in rodents—variations in inter-species susceptibility were reported by Valero et al. (2017), who needed around 20 dB higher noise level to induce a similar degree of cochlear synaptopathy in primates compared to rodents—and therefore, it could be the case that the actual noise-induced neurophysiological damage in humans is minimal. Another possible explanation is that the existing measures (mostly relying on ABR and EFR measures) are not sensitive enough to the neurophysiological damage associated with HHL, and that large inter-subject variability in these measures prevents their use in selectively diagnosing underlying neurophysiological pathologies at the individual level (Valderrama et al., 2018; Bramhall et al., 2019). In fact, current measures based on ABR, EFR and MEMR are affected by several extraneous factors, such as hair-cell loss in basal regions of the cochlea (Don and Eggeront, 1978; Yeend et al., 2017), ear canal effects that add variability to the auditory stimulus presented in testing, even if an insert earphone is used (Souza et al., 2014), and individual variance in the spectral component of MEMR measurements—which could compromise sensitivity when a tone probe is used to measure the MEMR (Bharadwaj et al., 2019). Further, considering that noise exposure accelerates the effects of aging (Fernandez et al., 2015), it is possible that young adults with a history of noise exposure have not yet developed substantial degradation of inner-hair cell synapses or demyelination. This would explain the negative results reported by several studies conducted in young adults (Prendergast et al., 2016; Fullbright et al., 2017; Grinn et al., 2017; Guest et al., 2019). It should also be noted that regardless of the metric, estimates of noise-exposure history are unvalidated and largely subjective, and range from estimates made over recent years to estimated noise-exposure history over the lifetime (Valderrama et al., 2018; Bramhall et al., 2019).

## Intervention strategies for hidden hearing loss

Interventions strategies for HHL can be classified in two categories: assistive listening devices that improve the hearing experience of their users, and emerging therapeutic interventions aimed at restoring the neurophysiological damage.

### Assistive listening devices

In the absence of any definitive objective measure or diagnostic for HHL in humans, researchers and clinicians continue to rely on questionnaires and surveys, to ascertain the hearing difficulties associated with HHL and to suggest treatment options. Koerner et al. (2020), for example, reported that, in addition to counseling patients with tactics that improve communication in noisy venues, around 23% of surveyed audiologists (*n* = 157) used mild-gain hearing aids as their preferred rehabilitation strategy, even though little-to-no research has been conducted to evaluate the efficacy of these technologies in adults with hearing difficulties but normal audiograms. In fact, to date, only two studies have investigated the use of a mild-gain hearing aid for this population (Roup et al., 2018; Singh and Doherty, 2020). These studies showed that while mild-gain hearing aids helped people with HHL reduce their hearing-in-noise handicap to some extent, only 3 out 17 participants in Roup et al. (2018), and 2 from 10 participants in Singh and Doherty (2020), reported being willing to continue using the devices in noisy listening situations. These studies suggest that whilst mild-gain hearing aids might potentially reduce the hearing-in-noise handicap of individuals with normal hearing, these technologies remain suboptimal for most of them. Another possible explanation for the low uptake of such technologies might be related to the effects of compression and amplification algorithms—a neurophysiological study conducted in hearing-impaired gerbils showed that although these algorithms help improve sound perception, they fail to restore the selectivity of neural responses to different speech sounds (Armstrong et al., 2022).

### Therapeutic interventions for synaptopathy

If synaptopathy represents a primary lesion in HHL, it makes sense to target the inner ear with therapeutics that might ameliorate its effects or reverse it altogether. Neurotrophins are a family of proteins that participate in the development and growth of neurons (Reichardt, 2006), and have been used to investigate the regeneration of the neurophysiological damage associated with cochlear synaptopathy. Wise et al. (2005) found in drug-induced deaf guinea pigs, that spiral ganglion cells regenerated peripheral axons of auditory nerve fibers toward their target inner hair cell following a cochlear perfusion of neurotrophin-3. Further, Suzuki et al. (2016) reported that round-window delivery of neurotrophin-3 24 h following exposure to a synaptopathic noise insult regenerated a significant proportion of the lost synaptic connections in mice, and led to the recovery of the suprathreshold amplitude of the ABR wave I. In a more complete form of hearing loss, gene transfer into the inner ear of guinea pigs deafened with gentamicin and implanted with cochlear implants demonstrated the capacity not only to grow neurites back toward potential targets using neurotrophins, but to use the electrode contacts within the ear to steer the therapy toward the desired location (Pinyon et al., 2019). These results support that a therapeutic intervention based on neurotrophins has the potential to prevent, decelerate or restore the adverse effects of cochlear synaptopathy in humans.

## Discussion

### Toward sensitive diagnostic biomarkers of hidden hearing loss in humans

Despite animal studies provide solid models of neurophysiological pathologies plausibly involved in HHL, research efforts inspired by Kujawa and Liberman’s (2009) seminal study of synaptopathy have, to date, failed to identify non-invasive biomarkers of HHL in humans appropriate for diagnostic purposes. Here, we discuss considerations and promising research opportunities provided by emerging methodological tools that seek to overcome barriers to the identification of non-invasive biomarkers of HHL.

One factor in the failure to identify HHL in human listeners is the aim to “hunt for pure HHL” which likely exists only rarely, if at all, beyond experimental laboratory settings. If cochlear synaptopathy precedes damage to outer hair cells (Kujawa and Liberman, 2009), most incidents of HHL are likely comorbid with “standard” audiometric hearing loss, especially given the near decade delay between individuals (or their associated others) noticing they might have hearing problems and seeking professional help (Simpson et al., 2019). To this end, one group of listeners for whom HHL is almost certainly an issue are those with near-normal thresholds or mild hearing loss.

Another barrier to developing biomarkers for HHL is a continued focus on cochlear synaptopathy, ignoring the role of other pathologies that might also underlie speech-in-noise difficulties reported by individuals with normal audiograms. Future efforts might usefully focus on developing novel non-invasive biomarkers that also target auditory nerve demyelination, central gain, and mal-adaptation. An example of such a biomarker might be an objective metric of performance in binaural listening tasks such as the *interaural phase modulation—following response* (IPM-FR, Undurraga et al., 2016). Since the neural encoding of small interaural time differences requires exquisite temporal precision in the activity of the auditory nerve from both ears (Stange-Marten et al., 2017), problems arising from demyelination might be expected to degrade this measure (Resnik and Rubinstein, 2021). Indeed, Bernstein and Trahiotis (2016) reported that individuals with highly sensitive hearing thresholds at 4 kHz (better than 7.5 dB hearing level) but reporting problems listening in noise performed worse in a binaural behavioral task, suggesting that early signs of hearing loss might be associated with deficits in binaural listening. Further, a study conducted on 23 normal-hearing listeners demonstrated a strong correlation between the amplitude of the IPM-FR and the ability to understand speech in noise as a function of interaural-time differences resulting from the spatial location of the speaker, both at individual and group levels, supporting the potential sensitivity of this measure to speech-in-noise hearing difficulties expected in individuals with HHL (Undurraga et al., 2020).

**FIGURE 3:**
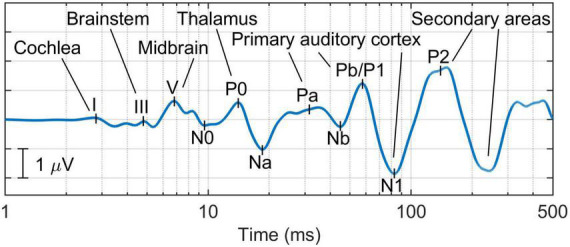
Example of the *full-range* auditory evoked response, which provides a comprehensive representation of all the components of the auditory pathway—from the cochlea to the cortex (de la Torre et al., 2020).

Additionally, new biomarkers could be retrieved from the *full-range* auditory evoked response (see Figure 3; de la Torre et al., 2020)—this response applies a latency-dependent filtering which, combined with the representation of the signal in the logarithmic time scale, enables the representation of all the components of the auditory pathway, from cochlea to cortex. This novel representation of transient auditory evoked potentials not only provides standard metrics such as the amplitude of wave I [appropriate to study synaptopathy (Kujawa and Liberman, 2009)], the waves I-III interpeak latency [since demyelination impairs the neural transmission time in the auditory nerve, longer values in this metric could be associated with demyelination this pathology (Wan and Corfas, 2017)], and the waves I-V amplitude ratio—an index of elevated central gain in the midbrain (Schaette and McAlpine, 2011); but also novel relative measures between central and peripherical components such as the ratio of the amplitude of wave I to P1 to assess the presence of elevated cortical gain proposed by Resnik and Polley (2021).

**FIGURE 4:**
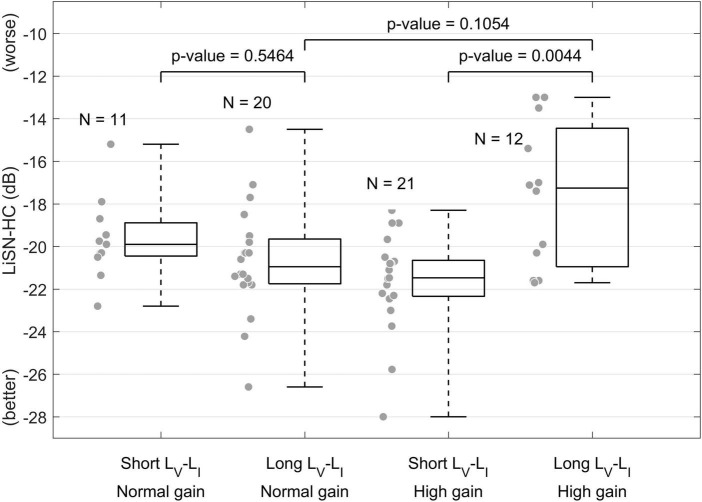
Effect of elevated central gain and brainstem neural transmission time on speech-in-noise intelligibility (Valderrama et al., 2018). This figure shows that when central gain at the level of the midbrain is elevated (high gain), individuals with longer brainstem neural transmission time (measured *via* the ABR waves I-V interpeak latency) presented worse speech-in-noise hearing performance.

The potential sensitivity of these measures to diagnosing problems listening in noise is supported by recent data from our own research. Figure 4 presents speech-in-noise hearing performance measured *via* the high-cue (HC) condition of the Listening in Spatialized Noise test (LiSN, Cameron and Dillon, 2008) on a cohort of 64 individuals with normal audiograms reporting different degrees of hearing-in-noise difficulties, who were categorized according to whether they had elevated central gain and their brainstem neural transmission time (measured *via* the ABR waves I-V amplitude ratio and interpeak latency, respectively) (Valderrama et al., 2018). These data demonstrate that in individuals with elevated central gain, those with longer brainstem neural transmission times showed impaired speech-in-noise performance, demonstrating an interaction between neural conduction times and elevated central gain. Consequently, it is reasonable to hypothesize that biomarkers associated with elevated central gain and neural transmission times might help characterize speech-in-noise intelligibility difficulties in individuals with normal audiograms.

A second barrier is the technical limitation imposed by standard processing methods that average several segments of the electroencephalogram to increase the signal-to-noise ratio (SNR) of the auditory evoked response. These traditional methods impose important constraints on the experimental design to meet the requirement of the inter-stimulus interval being longer than the duration of the evoked response so that the estimation of one response is not affected by adjacent responses (Valderrama et al., 2012). Overcoming this problem requires signal-processing algorithms that enable deconvolution of overlapping auditory evoked potentials. Some examples of these algorithms are iterative randomized stimulation and averaging (IRSA, Valderrama et al., 2014, 2016; de la Torre et al., 2019) and subspace-constrained least squares deconvolution (SC-LS, de la Torre et al., 2022). Importantly, IRSA enables the recording of the full-range response evoked by the fine structure of natural speech (Valderrama et al., 2019), and therefore provides a novel measure that may help advance knowledge in how the human auditory system encodes speech in challenging listening scenarios—a critical step to characterize HHL with objective biomarkers.

Another barrier to understanding HHL is the use of non-invasive methodologies in human research—which presents a *validation* problem (Plack et al., 2016) because it is difficult to assess *pre-mortem* human neural structures and confirm that a diagnostic tool is sensitive to a certain pathology. As a substitute, pathologies and biomarkers might be simulated in computational models of the auditory system. Examples of this approach include validation of the wave I of the ABR as a biomarker for cochlear synaptopathy (Verhulst et al., 2016), simulation of demyelination on the neural encoding of interaural time differences (Resnik and Rubinstein, 2021), characterization of the combined effect of synaptopathy and demyelination on the compound action potential (Budak et al., 2021), and simulation of the effect of different hearing damage mechanisms on speech-in-noise perception (Haro et al., 2020). Simulating pathologies in a computational model provides a controlled environment with the opportunity to validate the sensitivity of novel biomarkers to the target pathologies.

An important consideration is that even if a specific measure is found to be sensitive to HHL at group level (e.g., presenting statistically significant differences between the distributions observed on an *experimental* group with HHL and a *control* group with no hearing problems), the large inter-subject variability typically observed in neurophysiological measures such as metrics derived from the ABR or the EFR might prevent their use for diagnostic purposes (see Figure 4). One approach that has been reported to overcome this problem is the use of relative measures such as amplitude ratios, inter-peak latencies, or the slope of growth functions. The use of relative measures will likely rule out individual effects that add variability to specific measures, e.g., head size, ear canal shape, the individual anatomy of cochlear mechanics (Bharadwaj et al., 2019). A second approach is to analyze multiple biomarkers targeting different neurophysiological pathologies, through a comprehensive test battery of electrophysiological, behavioral, cognitive, and psychoacoustic measures, and use machine learning to estimate the magnitude of hearing damage associated with HHL. Machine learning approaches have been used to predict noise-induced hearing impairment in individuals exposed to complex industrial noise (Yanxia et al., 2019), and could provide links between neurophysiological pathologies and perceptual difficulties, essential to developing a sensitive diagnostic tool for HHL.

A common problem faced by most investigations of HHL in humans is the validity of estimates of noise-exposure history, as these rely heavily on subjective questionnaires and self-reported measures. In this respect, future investigations could benefit from emerging technologies such as portable noise-exposure dosimeters embedded in wearables like smart watches to generate more reliable measures of noise exposure. Further, access to individualized metrics of noise exposure background may also benefit from *citizen science* or crowd-sourcing of data, an ideal means of identifying individuals at risk of HHL, tailoring strategies to prevent hearing loss, and engaging beneficiaries of future therapies and interventions for HHL. An example of this approach is the Apple Hearing Study (Apple Inc., Cuppertino, CA)—a large-scale national study conducted in the United States that uses mobile applications on the Apple Watch to assess the intensity of environmental sounds and cardiovascular metrics in order to understand the impacts of being exposed to loud sounds on hearing and cardiovascular health (Neitzel et al., 2022).

A final consideration relies on the methodologies used to measure the MEMR—a potential biomarker for HHL in humans (Valero et al., 2016, 2018; Wojtczak et al., 2017). Although standard clinical measures of the MEMR employ pure tones (typically at 226 Hz or 1000 Hz) to evoke the reflex (Schairer et al., 2013), Bharadwaj et al. (2019) suggested that individual variations in the middle-ear anatomy may influence the frequency spectrum and magnitude of MEMR measures. This might explain the null results reported by Guest et al. (2019) when they investigated the relationship between MEMR and tinnitus, speech-in-noise performance, and noise-exposure background. Wideband probe stimuli such as chirps or clicks could be used as probe stimuli to overcome this problem and increase sensitivity. Novel MEMR methodologies based on click-evoked otoacoustic emissions such as the one developed by Boothalingam et al. (2021) could play an important role in the differential diagnosis of HHL.

### On the search of optimal management strategies

While therapeutic interventions may eventually prevent the start, delay the progression of, or even reverse, the impairment of age- and noise-induced HHL, it will likely be some time before clinicians can administer an efficient drug to treat patients with HHL. This means that there is an urgent need to develop and standardize a non-pharmacologic solution that improves the hearing experience of individuals with HHL.

An immediate approach to help HHL patients to deal with their hearing difficulties could be to provide them with training on coping strategies typically used by people with hearing loss, e.g., mobile- and web-based applications that provide lipread training. Pang et al. (2019) identified that the most commonly reported coping strategies used by HHL individuals were non-verbal cues such as lip reading, gestures and facial expressions; moving closer or tilting toward the speaker; moving to quieter locations; concentrating harder in conversations; avoiding noisy places; and whenever possible, reducing the level of noise ambience, e.g., by turning down the television volume. Appropriate counseling about these coping strategies in clinical appointments could provide practical guidelines to HHL patients.

Edwards (2020) proposed a model anticipating which technologies would be preferred to attend the hearing needs of different segments of the broad spectrum of people with hearing difficulties. This model predicted that individuals who self-perceive hearing difficulties but do not have a measurable hearing loss are potential candidates for *hearables*—technologies that use directionality and smart audio processing to attenuate the effect of background noise and enhance the hearing experience of their users. In fact, a study conducted by the authors and their research teams showed that a significant proportion of individuals with speech-in-noise intelligibility difficulties but normal or near normal audiograms reported to be *ready and willing* to trial hearables in acoustically challenging situations such as cafeterias and noisy restaurants (Mealings et al., 2020). To this end, future research might usefully assess the value of hearables in meeting the unique hearing needs of individuals with HHL.

In order to validate the value of these technologies as an intervention for listening problems associated with HHL, clinicians need to know (i) to what extent these devices improve the hearing experience of their users [this will help clinicians manage the expectations of their patients]; (ii) what are the listening scenarios in which devices perform best/worse [this will help them provide adequate counseling on the capabilities of the devices]; (iii) what proportion of users benefit when using these devices in acoustically challenging situations [this will provide an estimation of the success rate of this intervention]; (iv) what are the unique features that characterize those who do benefit from these technologies [this will help clinicians anticipate which patients would benefit the most]; (v) how close do these listening devices match a prescription target [this will ensure users receive optimal audibility and that their hearing is not compromised as a consequence of any possible over-amplification]; and (vi) what are the main barriers that would discourage users from using the devices (e.g., cost, stigma, comfort, battery life) [this will help clinicians provide informed recommendations to their patients, and may also inspire technology manufacturers in the development of the next-generation products that will close the gap between the technology features and the users’ *gains* and *pains*, thus eventually increasing the adoption rate of these technologies]. Addressing these questions will likely lead to the development of clinical-management guidelines for HHL that could be standardized globally.

Unlike traditional research methods—largely based on laboratory-based measures of hearing and speech-in-noise intelligibility, novel methodologies based on *ecologically-momentary assessment* (EMA, Timmer et al., 2018) have the potential to capture difficult-to-assess factors such as user satisfaction, emotional state and perceived hearing benefit from listening technologies. In contrast to traditional questionnaires, which are usually applied at the completion of a study, EMA tools increase reliability and reduce recall bias by enabling users to provide real-time feedback of their hearing experience in those listening settings in which they experience difficulties. In addition, EMA tools can record acoustic features of the sound environment such as the A-weighted sound level and reverberance, which would help respond to some of the research questions mentioned above. Further, future research methodologies might also consider conducting a randomized control trial, including a control group fitted with an *acoustically transparent* device (i.e., a hearing device that does not apply any gain or compression) to account for any possible placebo effect derived from device placement within the ear (Dawes et al., 2013).

The use of assistive devices such as hearables to improve the listening experience of people with HHL involves providing users with a mild gain (i.e., 5–10 dB insertion gain) that may compensate for some degree of hearing loss, and provides an acoustic advantage in noisy environments thanks to the directionality of their microphones and noise-reduction algorithms. Future research endeavors might investigate whether the acoustic benefit of these devices increases by incorporating advanced signal-processing features that have been proven successful in hearing aids and cochlear implants, including adaptive selection of the device output levels to optimally fit an individual’s hearing dynamic range (Blamey, 2005), smart algorithms based on contralateral inhibition that enhance binaural cues (Lopez-Poveda et al., 2022), and the use of effective voice activity detection algorithms that provide an enhanced SNR to the listener in situations with background noise (Ramirez et al., 2004; de la Torre et al., 2006; Liu and Demosthenous, 2021).

**FIGURE 5:**
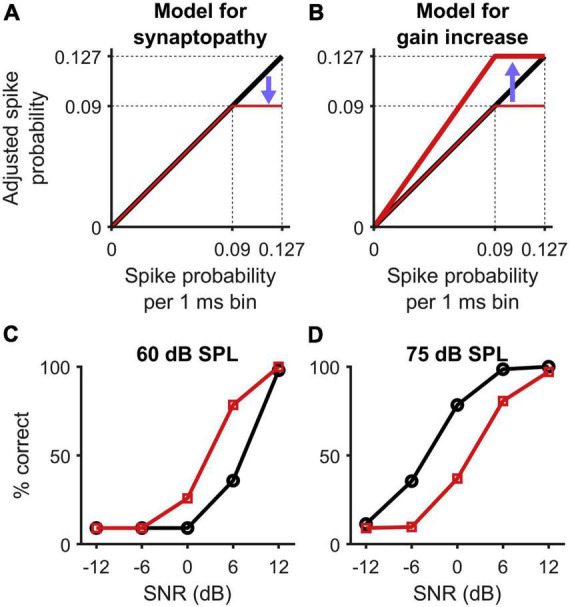
Figure adapted from Monaghan et al. (2020) presenting a model for synaptopathy and central gain activation. **(A)** The loss of high-threshold auditory nerve fibers in cochlear synaptopathy saturates the spiking probability of neurons in the inferior colliculus at supra-threshold level. **(B)** Central gain activation presents a multiplicative increase of the neurons sensitivity to restore the maximum (non-synaptopathic) spike probability. **(C,D)** As a consequence, the slope of the spike probability function increases in mid-levels, which leads to better discriminability from the HHL model (squares) than from the control model (circles) at 60 dB SPL, but reduced discriminability at 75 dB SPL.

Other avenues to explore include novel interventions based on attenuating (rather than amplifying) high-intensity sounds. The intention of this apparently counter-intuitive approach is to shift input sounds to a level range in which individuals with HHL are expected to have optimal sensitivity. Recent experimental animal findings [see Figure 5, adapted from Monaghan et al. (2020)] suggest that loss of, or damage to, high-threshold auditory nerve fibers resulting from cochlear synaptopathy leads to a saturation of the spiking probability in neurons of the inferior colliculus (Figure 5A). Elevation of central gain (Figure 5B) in response to reduced sensory input seeks to restore the maximum (non-synaptopathic) spike probability, with the consequence that the slope of the spike-probability function increases for mid-level sounds, leading to better discriminability in the HHL model relative to that in the control model at 60 dB SPL, but not at 75 dB SPL (Figures 5C,D). Based on this model, an assistive listening device based on an attenuator could potentially help individuals with HHL communicate better in noisy and loud environments.

Finally, it could be the case that a *one-size-fits-all* solution is not appropriate to reducing listening difficulties associated with HHL, and that different solutions are required for different segments of the HHL population. In this regard, the objective determination of the site of neurophysiological lesion might lead to the development of different strategies tailored to individual listeners. For example, technologies based on directionality or background-noise reduction might improve the hearing experience of individuals with synaptopathy; technologies that enhance binaural-hearing cues such as binaural-weighted subtraction (Lopez-Poveda et al., 2022) could help individuals with auditory nerve demyelination problems; and cognitive training programs could benefit individuals with intact peripherical neural structures but with selective-attention difficulties. The use of objective methods that provide measures from both peripherical and central neural stations such as the full-range auditory evoked potential (de la Torre et al., 2019) could help identify neural structures presenting abnormal activity patterns.

## Authors contributions

JV conceived and wrote the manuscript. AT and DM provided the conceptual framework, assisted in the literature review, and participated in the writing of the manuscript. DM proofread the English of the manuscript. All authors read and approved the final manuscript.
